# Effects of Rotary Tillage and Fertilization on Chemical Properties and Microbial Communities of Soil Under Continuous *Morchella* Mushroom Cultivation

**DOI:** 10.3390/biology15090674

**Published:** 2026-04-24

**Authors:** Wei Qi, Litao Lü, Kai Huang, Jianzhao Qi, Minglei Li, Mingwen Shi, Hong Wang

**Affiliations:** 1 Institute of Edible Fungi, Liaoning Academy of Agricultural Sciences, Shenyang 110000, China; 2 School of Soil and Water Conservation Science and Engineering, Northwest A&F University, Yangling 712100, China; 3 Center of Edible Fungi, Northwest A&F University, Yangling 712100, China; qjz@nwafu.edu.cn; 4 Shaanxi Key Laboratory of Natural Products & Chemical Biology, College of Chemistry & Pharmacy, Northwest A&F University, Yangling 712100, China

**Keywords:** *Morchella*, continuous cropping obstacle, calcium cyanamide, soil microorganisms, metagenomics, soil chemical properties

## Abstract

The severe continuous cropping obstacles in *Morchella* cultivation, driven primarily by soil microecological imbalance, critically constrain the sustainable development of the industry. To address this challenge, this study evaluated the efficacy of rotary tillage, calcium cyanamide (CaCN_2_), and organic fertilizer, applied individually and in combination, in mitigating these obstacles and explored the underlying microbial mechanisms. The soil was treated on 5 August 2024, and soil samples were collected on 5 October 2024. Four treatments were established: continuous cropping control (CK), rotary tillage (XGX), rotary tillage combined with calcium cyanamide (MPD), and rotary tillage combined with calcium cyanamide and organic fertilizer (MPX). Soil chemical properties were analyzed in conjunction with metagenomic sequencing to characterize the responses of soil properties and microbial communities, including both eukaryotic and bacterial taxa. The results indicated that the MPD treatment showed a relatively pronounced effect in enhancing key soil fertility indicators, including soil organic matter (OM), total nitrogen (TN), available nitrogen (AN), available potassium (AK), and total phosphorus (TP). All amendments significantly altered microbial community structures. Specifically, the integrated MPX treatment effectively reduced the relative abundance of the pathogenic fungus *Olpidium* while maintaining higher overall microbial diversity. It also significantly promoted the abundance of *Morchella* itself and beneficial bacterial phyla such as Actinomycetota and *Pseudomonadota*. Redundancy analysis identified AN and AK as the primary drivers of eukaryotic community variation, whereas Availa-ble phosphorus (AP) and potential of hydrogen (pH) were the key factors shaping the bacterial community. The results indicated that MPD was the showed relatively pronounced effectiveness in rapidly improving soil fertility and suppressing pathogenic fungi. In contrast, MPX showed relatively better performance in optimizing microbial community structure, enhancing microbial diversity, and strengthening overall ecological stability. These two treatments exhibited distinct advantages in soil chemical improvement and microbial community regulation, respectively, thereby providing alternative practical strategies and a theoretical basis for the ecological management of continuous-cropping obstacles in *Morchella* cultivation. It should be noted that this study did not include treatments with calcium cyanamide alone, organic fertilizer alone, or their combined application without rotary tillage. This is primarily because rotary tillage is a standard land preparation practice in *Morchella* cultivation, and the use of soil amendments without accompanying tillage is rarely adopted under practical production conditions.

## Simple Summary

*Morchella* is a high-value edible fungus; however, continuous cropping leads to declining yields and heightened disease incidence—a phenomenon termed the continuous cropping obstacle. This study employed field trials to evaluate the efficacy of various combinations of rotary tillage, calcium cyanamide, and fertilizer in ameliorating soils affected by this constraint. The results demonstrated that the integrated application of rotary tillage, calcium cyanamide, and organic fertilizer most effectively enhanced soil quality. This regimen not only elevated soil nutrient levels but also optimized the microbial community structure—suppressing pathogenic fungi whilst fostering beneficial microorganisms. These findings indicate that this holistic management strategy alleviates continuous cropping impediments in morel cultivation, thereby offering practical guidance for the sustainable development of the edible mushroom sector.

## 1. Introduction

*Morchella* belongs to the phylum *Ascomycota*, class *Pezizomycetes*, and order *Pezizales*, and is a rare edible fungus of significant economic value. Its fruiting body is named for its distinctive honeycomb-like cap, resembling a sheep’s stomach [1,2] and it is rich in nutritional value [3,4,5]. In recent years, the Morchella cultivation industry in China has undergone rapid expansion. The production area has extended from a few provinces to over 20 across the country, with cultivation models becoming increasingly diversified. This growth has established *Morchella* cultivation as a characteristic industry contributing to rural revitalization. However, alongside the continuous expansion of cultivation scale, the challenge of continuous cropping obstacles has become increasingly pronounced. These obstacles are typically manifested as a significant decline in yield and a marked increase in disease and pest incidence after two or more consecutive planting cycles. This issue has emerged as a key constraint on the sustainable and healthy development of the industry.

The occurrence of continuous cropping obstacles in *Morchella* cultivation is primarily associated with two factors. First, the continuous accumulation of autotoxic substances in the soil directly inhibits mycelial growth and fruiting body development. Second, soil microecological imbalance leads to the proliferation of pathogenic microorganisms and the decline of beneficial microbial communities [6,7]. In *Morchella* cultivation, conventional practices such as rotary tillage and disinfection with quicklime are commonly used for soil management. However, under continuous cropping conditions, problems such as soil microecological imbalance, proliferation of pathogenic microorganisms, and accumulation of autotoxins have not yet been effectively resolved [8]. At present, research on effective strategies to mitigate continuous cropping obstacles is still relatively limited. Although some studies have reported that appropriate crop rotation systems can help maintain the balance of soil microbial communities [9], their implementation is often constrained by land availability in large-scale and specialized production systems. Therefore, the development of integrated agronomic practices that can effectively improve soil chemical properties and regulate microbial community structure is of great importance for achieving sustainable *Morchella* cultivation. Tillage practices and soil amendments are key approaches for regulating soil properties. Rotary tillage can alter soil physical structure through mechanical disturbance, thereby influencing microbial habitats [10,11]. CaCN_2_, as a multifunctional soil amendment, undergoes hydrolysis in the presence of soil moisture to produce H_2_CN_2_, which is subsequently transformed into urea and further mineralized into ammonium nitrogen. This transformation pathway can temporarily inhibit soil nitrification, thereby reducing nitrate leaching and improving nitrogen use efficiency. In addition, the decomposition of calcium cyanamide releases calcium ions, which exert a liming effect and contribute to the regulation of soil pH. Collectively, these processes not only enhance soil chemical properties but also create a more favorable environment for microbial activity [12,13]. In addition, the application of organic fertilizers can improve soil aggregate structure and enhance nutrient retention capacity, thereby creating a favorable environment for the growth of beneficial microorganisms [14,15].

Based on the above theoretical framework, we hypothesized that the combined application of rotary tillage, calcium cyanamide, and organic fertilizer could effectively alleviate continuous-cropping obstacles in *Morchella* cultivation by improving soil properties and reshaping a healthy microbial community structure. To test this hypothesis, four treatments were established: continuous cropping control, rotary tillage alone, rotary tillage combined with calcium cyanamide, and rotary tillage combined with calcium cyanamide and organic fertilizer. These treatments were selected to separately evaluate the physical regulatory effects of rotary tillage, the chemical disinfection and fertility enhancement provided by calcium cyanamide, and the synergistic role of organic fertilizer in modulating microbial communities. By comparing differences in soil physicochemical properties, as well as the diversity and composition of eukaryotic and bacterial communities among treatments, and by identifying key environmental factors driving microbial community variation, this study aimed to elucidate the mechanisms by which different agronomic practices mitigate continuous-cropping obstacles in *Morchella* cultivation, thereby providing a theoretical basis and technical support for the ecological restoration of continuously cropped soils.

## 2. Materials and Methods

### 2.1. Overview of Experimental Site and Materials

The experiment was conducted from August 2024 to April 2025 at a *Morchella* cultivation base of the Institute of Edible Fungi, Liaoning Academy of Agricultural Sciences, located in Shenyang City, Liaoning Province, China. Studies on Calcium Cyanamide. I. The Decomposition of Calcium Cyanamide in the Soil and its Effects on Germination.

The tested soil was classified as brown soil (Haplic Luvisol, according to the World Reference Base for Soil Resources, WRB). The previous crop was *Morchella*, which had been continuously cultivated for three consecutive years. With increasing years of continuous cultivation, the yield of *Morchella* exhibited a progressive decline; by the third year, yields had decreased by more than 60% compared to the first year, and complete crop failure was observed in some plots, indicating typical symptoms of continuous cropping obstacles.

### 2.2. Experimental Design

Four treatments were established in this study: (1) CK, continuous cropping control, in which the original cultivation practice was maintained without any intervention; (2) XGX, rotary tillage treatment, with tillage performed twice at a depth of 15 cm on 5 August and 5 September 2024; (3) MPD, rotary tillage combined with calcium cyanamide, in which calcium cyanamide (CaCN_2_, ≥55% active ingredient) was applied at 600 kg ha^−1^ on 5 August 2024, followed by immediate rotary tillage, and a second tillage was conducted on 5 September 2024; and (4) MPX, rotary tillage combined with calcium cyanamide and organic fertilizer, in which calcium cyanamide (600 kg ha^−1^) was applied on 5 August 2024, followed by immediate rotary tillage, and well-decomposed sheep manure organic fertilizer (organic matter ≥ 45%) was applied at 7500 kg ha^−1^ on 5 September 2024, followed by immediate rotary tillage.

Soil samples from all treatments were collected on 5 October 2024. Each treatment was replicated three times using a randomized complete block design. Based on the topography and soil texture distribution of the experimental field, the 12 plots were divided into three blocks, with each block containing four plots corresponding to the four treatments. The treatments were randomly assigned within each block. Each plot covered an area of 12 m^2^ (4 m × 3 m), with a spacing of 0.8 m between adjacent plots.

### 2.3. Soil Sample Collection

Soil treatments were applied to the experimental plots on 5 September 2024. Soil samples were collected one month later, on 5 October 2024, from the 5–15 cm soil layer in each plot using a five-point sampling method. Each composite sample was immediately divided into two subsamples. One subsample was placed in an icebox, transported to the laboratory, and stored at −80 °C for subsequent microbial community analysis. The other subsample was air-dried at room temperature, homogenized, and passed through a 2 mm sieve for the analysis of soil chemical properties (Figure S1).

### 2.4. Analytical Methods

#### 2.4.1. Soil Chemical Properties

Soil pH (potential of hydrogen) was measured potentiometrically in a 1:2.5 (*w*/*v*) soil–water suspension. OM content was determined by the potassium dichromate external heating method. Total nitrogen (TN) was quantified using the Kjeldahl method. Available nitrogen (AN) was assessed by the alkaline diffusion method. Total phosphorus (TP) was measured following sodium hydroxide fusion and molybdenum–antimony colorimetry. Available phosphorus (AP) was extracted with sodium bicarbonate and determined by molybdenum–antimony colorimetry. Total potassium (TK) and available potassium (AK) were analyzed by flame photometry.

#### 2.4.2. Microbial Community Analysis

Soil DNA was extracted using the Mag-Bind^®^ Soil DNA Kit (Omega Bio-tek, Norcross, GA, USA) according to the manufacturer’s instructions. DNA concentration and purity were assessed using a NanoDrop 2000 spectrophotometer (Thermo Fisher Scientific, Waltham, MA, USA), and integrity was evaluated by 1% agarose gel electrophoresis. The extracted DNA was fragmented using a Covaris M220 system (Gene Company Limited, Shanghai, China), and fragments of approximately 350 bp were selected for library construction. Paired-end libraries were prepared using the NEXTFLEX Rapid DNA-Seq Library Preparation Kit (Bioo Scientific, Austin, TX, USA). After quality assessment, the libraries were subjected to metagenomic sequencing on an Illumina NovaSeq 6000 (Illumina, San Diego, CA, USA) platform (Majorbio Bio-Pharm Technology Co., Ltd., Shanghai, China). Three biological replicates were included for each treatment (*n* = 3), resulting in a total of 12 samples for sequencing analysis. Raw sequencing data were quality-filtered using fastp (v0.23.0) to remove adapter sequences and low-quality reads (average base quality score < 20), retaining high-quality reads with lengths ≥ 50 bp. Host-derived contaminant reads were removed by aligning the reads to the host DNA sequences using BWA (v0.7.17). The clean reads were then assembled into contigs using MEGAHIT (v1.2.9), and contigs with lengths ≥ 300 bp were retained for subsequent analyses. Open reading frames (ORFs) were predicted from the contigs using MetaGene (v3.8), and genes with nucleotide lengths ≥ 100 bp were selected and translated into amino acid sequences. A non-redundant gene catalog was constructed using CD-HIT (v4.8.1) with parameters of 90% sequence identity and 90% coverage. High-quality reads from each sample were mapped to the non-redundant gene set using SOAPaligner (v2.21) (95% identity) to obtain gene abundance profiles.

For taxonomic annotation, the amino acid sequences of the non-redundant gene set were aligned against the NR database using DIAMOND (v0.8.35) (BLASTP, e-value ≤ 1 × 10^−5^) to obtain taxonomic assignments and calculate species abundance. For functional annotation, the amino acid sequences were compared against the KEGG and eggNOG (COG) databases using DIAMOND (e-value ≤ 1 × 10^−5^) to assign functional categories and estimate their relative abundances.

### 2.5. Statistical Analysis

Data were organized using Microsoft Excel 2020. Differences among treatments were assessed using one-way analysis of variance (one-way ANOVA) followed by Duncan’s multiple range test for post hoc comparisons, with statistical significance set at *p* < 0.05, all conducted using SPSS 26.0. (IBM Corp., Armonk, NY, USA) Multiscale relative abundance visualization, α-diversity analysis, β-diversity evaluation (principal coordinates analysis, PCoA, based on Euclidean distance), and linear discriminant analysis effect size (LEfSe) were performed using the Majorbio Cloud platform. Redundancy analysis (RDA) was carried out using Canoco 5 (Microcomputer Power, Ithaca, NY, USA) to examine the relationships between soil microbial communities and environmental factors. Mantel tests and Pearson correlation analysis were performed to assess the associations between soil chemical properties and microbial community diversity. Column charts were generated using Origin 2021 (OriginLab, Northampton, MA, USA).

## 3. Result

### 3.1. Effects of Different Treatments on Soil Chemical Properties

**Figure 1:**
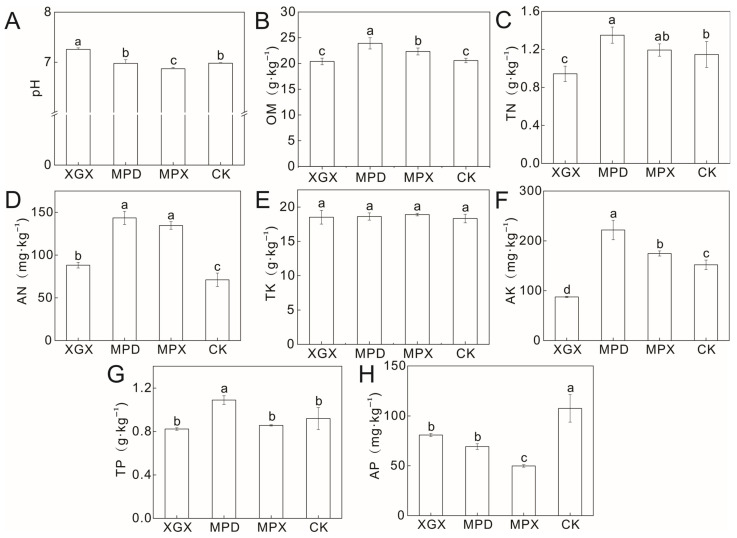
Content of soil chemical properties under different treatments. (**A**–**H**) shows the contents of soil pH, organic matter (OM), total nitrogen (TN), available nitrogen (AN), total potassium (TK), available potassium (AK), total phosphorus (TP), and available phosphorus (AP) in different treatments, with significant differences indicated by different lowercase letters (*p* < 0.05).

Soil chemical properties are key indicators of soil fertility and exert direct or indirect effects on crop yield and quality. In this study, eight soil chemical parameters were measured, including potential of hydrogen (pH), Organic Matter (OM), Total nitrogen (TN), Available nitrogen (AN), Available nitrogen (TK), Available potassium (AK), Total phosphorus (TP), Available phosphorus (AP). The results showed that the different treatments had significant effects on soil properties (Figure 1). Specifically, soil pH under the XGX treatment was significantly higher than that under the other treatments (Figure 1A). In terms of nutrient contents, the MPD treatment exhibited the highest levels of OM, TN, AN, AK, and TP, all of which were significantly different from those of the control (CK) (Figure 1B–D,F,G). The AN content in the XGX and MPX treatments was also significantly higher than that in CK (Figure 1D). No significant differences were observed in TK among the treatments (Figure 1E), whereas the AP content in CK was significantly higher than that in the other three treatments (Figure 1H). three amendment groups (Figure 1H).

### 3.2. Microbial Community Diversity Analysis

**Figure 2:**
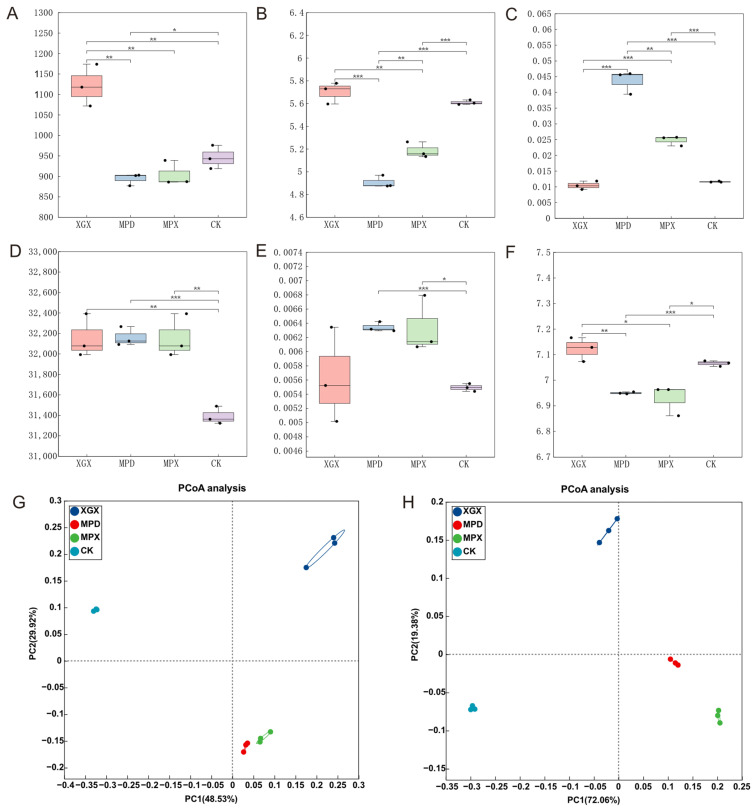
Microbial community diversity in different treatment groups. ACE index, Shannon index, and Simpson index of eukaryotic communities (**A**–**C**). ACE index, Shannon index, and Simpson index of bacterial communities (**D**–**F**). PCoA analysis of eukaryotic and bacterial communities (**G**,**H**). * (0.01 ≤ *p* < 0.05), ** (0.001 ≤ *p* < 0.01), *** (*p* < 0.001).

To assess the impact of different treatments on soil microorganisms, high-throughput sequencing was performed on soil samples. Raw sequencing data were quality-filtered using fastp. The results showed that the Q30 values (i.e., the proportion of reads with a base quality score ≥ 30) exceeded 92% for all samples (Table S1), indicating high sequencing quality and suitability for subsequent analyses. The results of α-diversity analysis indicated that different treatments had significant effects on soil microbial community diversity. In the eukaryotic community, both the ACE and Shannon indices under the XGX treatment were significantly higher than those in the control (CK) (Figure 2A,B), suggesting that rotary tillage increased species richness and evenness of eukaryotes. The Simpson indices in the MPX and MPD treatments were significantly higher than that in CK (*p* < 0.001). As the Simpson index reflects community dominance, with higher values indicating greater dominance of a few taxa, this result suggests that the application of calcium cyanamide may have suppressed certain sensitive groups, leading to a more simplified community structure. In the bacterial community, the ACE indices in all treatment groups were higher than that in CK, with the MPD treatment showing an extremely significant difference (*p* < 0.001). Meanwhile, the Shannon indices in the MPD and MPX treatments were also significantly higher than that in CK (Figure 2D–F). Overall, the MPX treatment exhibited a more pronounced effect on enhancing bacterial diversity. These findings indicate that eukaryotic and bacterial communities responded differently to the treatments, which may be attributed to differences in their sensitivity to environmental changes.

Beta diversity analysis based on PCoA showed clear separation among treatments. For eukaryotes, PC1 and PC2 explained 48.5% and 29.92% of the variance, respectively, while for bacteria, the corresponding values were 72.06% and 19.38%. Samples within each treatment group clustered tightly, whereas distinct separation was observed between groups (Figure 2G,H). This, together with Venn diagram analysis (Figure S2), demonstrates that the different soil amendments significantly altered the structure of the soil microbial community.

### 3.3. Microbial Community Compositional Variation Analysis

**Figure 3:**
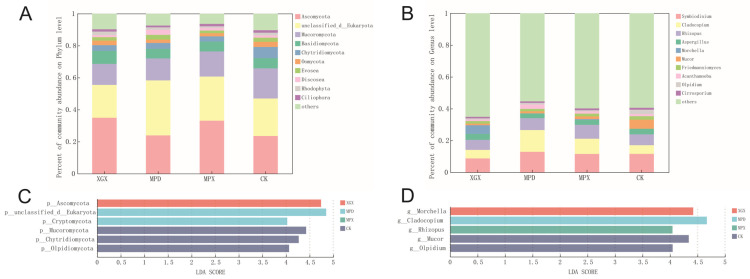
Analysis of eukaryotic community structure under different treatment conditions. Phylum-level and genus-level composition of eukaryotic communities in different treatment groups (**A**,**B**). Phylum-level and genus-level composition of eukaryotic Lefse in different treatment groups (**C**,**D**).

At the phylum and genus levels, the effects of different treatments on the composition of soil microbial communities were analyzed as follows: At the phylum level, the eukaryotic community was dominated by Ascomycota and *Mucoromycota* (Figure 3A). Ascomycota are important decomposers in soil, participating in the degradation of organic matter and nutrient cycling. *Mucoromycota* are primarily saprotrophic fungi that play a role in the decomposition of organic substrates. Multiple comparisons showed that all three treatments significantly increased the relative abundance of Ascomycota, while reducing the abundance of *Mucoromycota*, *Chytridiomycota*, and *Olpidiomycota* (Figure S3A). At the genus level (Figure S3B–D), all treatments significantly increased the relative abundance of *Morchella*, while significantly decreasing that of the pathogen-associated genus *Olpidium* (Figure 3B). LEfSe analysis further confirmed the significant differences in these taxa among treatments (Figure 3C,D). Figure 3A presents the top ten eukaryotic microbial phyla based on relative abundance, including Ascomycota, *Mucoromycota*, *Basidiomycota*, *Chytridiomycota*, *Oomycota*, *Evosea*, *Discosea*, *Rhodophyta*, and *Ciliophora* (average relative abundance ≥ 1%). Among these, taxa such as Rhodophyta were detected at relatively low abundances in soil and may originate from soil microalgae or algal residues derived from surface litter.

**Figure 4:**
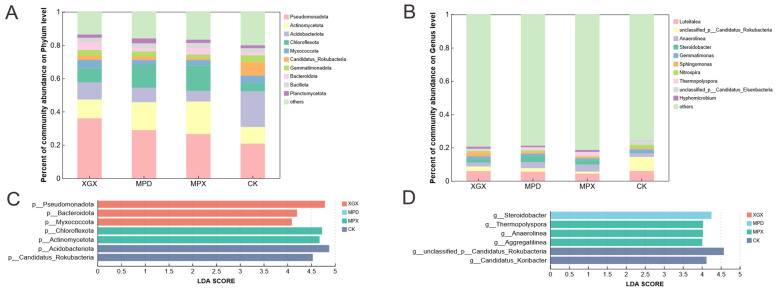
Analysis of bacterial community structure across different treatment groups. Phylum-level and genus-level composition of bacterial communities in different treatment groups (**A**,**B**). Phylum-level and genus-level characteristics of bacterial communities in different treatment groups (**C**,**D**).

In the bacterial community, at the phylum level, all three treatments significantly increased the relative abundance of *Pseudomonadota*, Actinomycetota, Chloroflexota, Bacteroidota, and Planctomycetota (Figure 4A and Figure S3C). At the genus level, taxa such as *Anaerolinea*, *Steroidobacter*, *Thermopolyspora*, and *Hyphomicrobium* were significantly enriched in the treatment groups (Figure 4B and Figure S3D). LEfSe analysis identified these taxa as key biomarkers distinguishing among treatments (Figure 4C,D).

### 3.4. Redundancy Analysis (RDA) of the Correlation Between Soil Chemical Properties and Microbial Community

**Figure 5:**
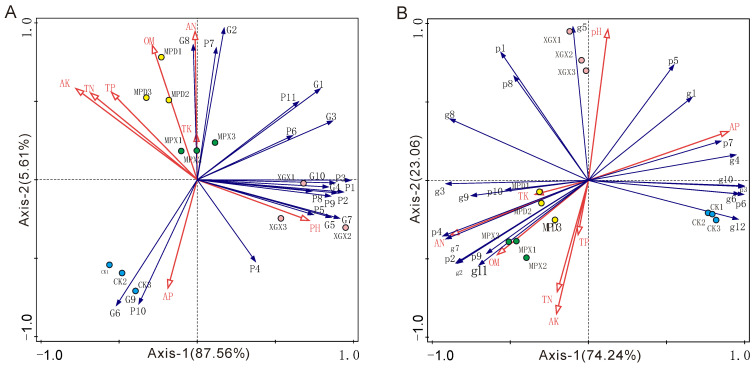
RDA of eukaryotic and bacterial communities. (**A**) The existing fungal taxa, P1: Ascomycota, P2: Mucoromycota, P3: Basidiomycota, P4: Chytridiomycota, P5: Oomycota, P6: Evosea, P7: Discosea, P8: Rhodophyta, P9: Ciliophora, P10: Olpidiomycota, P11: Cryptomycota, G1: *Symbiodinium*, G2: *Cladocopium*, G3: *Rhizopus*, G4: *Aspergillus*, G5: *Morchella*, G6: *Mucor*, G7: *Friedmanniomyces*, G8: *Acanthamoeba*, G9: *Olpidium*, G10: *Cirrosporium*. The existing bacterial taxa (**B**), p1: Pseudomonadota, p2: Actinomycetota, p3: Acidobacteriota, p4: Chloroflexota, p5: Myxococcota, p6: Candidatus_Rokubacteria, p7: Gemmatimonadota, p8: Bacteroidota, p9: Bacillota, p10: Planctomycetota, g1: *Luteitalea*, g2: *Anaerolinea*, g3: *Steroidobacter*, g4: *Gemmatimonas*, g5: *Sphingomonas*, g6: *Nitrospira*, g7: *Thermopolyspora*, g8: *Hyphomicrobium*, g9: *Streptomyces*, g10: *Candidatus*_*Solibacter*, g11: *Aggregatilinea*, g12: *Candidatus*_*Koribacter*.

The results showed that in the RDA of the eukaryotic community and environmental factors, the selected environmental variables explained 96.2% of the community variation (adjusted R^2^ = 86.0%), with the first two axes cumulatively accounting for 93.17% of the variation. AK and AN were identified as the primary environmental factors shaping the eukaryotic community, explaining 54.8% (*p* < 0.01) and 33.8% (*p* < 0.01) of the variation, respectively (Tables S2 and S3). In the RDA of the bacterial community and environmental factors, the selected variables explained 99.0% of the community variation (adjusted R^2^ = 96.4%), with the first two axes cumulatively explaining 97.3% of the variation. Forward selection analysis indicated that AP and pH were the key environmental factors driving changes in bacterial community structure, with marginal contributions of 62.2% (*p* < 0.01) and 25.2% (*p* < 0.01), respectively, to the total explained variation in the model (Tables S4 and S5, Figure 5).

**Figure 6:**
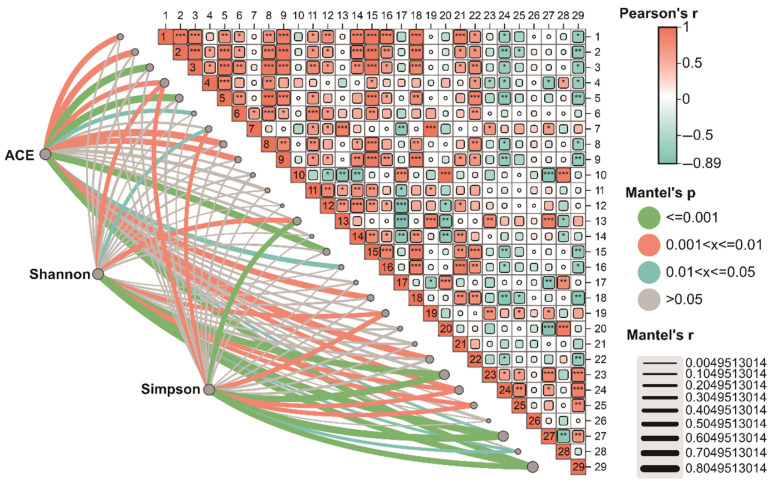
Mantel correlation analysis based on eukaryotic communities. 1: *Ascomycota*, 2: *Mucoromycota*, 3: *Basidiomycota*, 4: *Chytridiomycota*, 5: *Oomycota*, 6: *Evosea*, 7: *Discosea*, 8: *Rhodophyta*, 9: *Ciliophora*, 10: *Olpidiomycota*, 11: *Cryptomycota*, 12: *Symbiodinium*, 13: *Cladocopium*, 14: *Rhizopus*, 15: *Aspergillus*, 16: *Morchella*, 17: *Mucor*, 18: *Friedmanniomyces*, 19: *Acanthamoeba*, 20: *Olpidium*, 21: *Cirrosporium*, 22: pH, 23: OM, 24: TN, 25: TP, 26: TK, 27: AN, 28: AP, 29: AK. * (0.01 ≤ *p* < 0.05), ** (0.001 ≤ *p* < 0.01), *** (*p* < 0.001).

Spearman correlation analysis was further employed to identify specific microbial taxa significantly associated with these key soil properties. Within the eukaryotic community, AK showed significant negative correlations with several phyla, including *Ascomycota*, *Mucoromycota*, *Basidiomycota*, *Chytridiomycota*, *Oomycota*, *Rhodophyta*, and *Ciliophora*, as well as with the genera *Aspergillus*, *Morchella*, and *Friedmanniomyces*. In contrast, AN was significantly positively correlated with *Discosea*, *Cladocopium*, and *Acanthamoeba*, while exhibiting significant negative correlations with *Chytridiomycota*, *Olpidiomycota* and *Rhizopus* (Figure 6).

**Figure 7:**
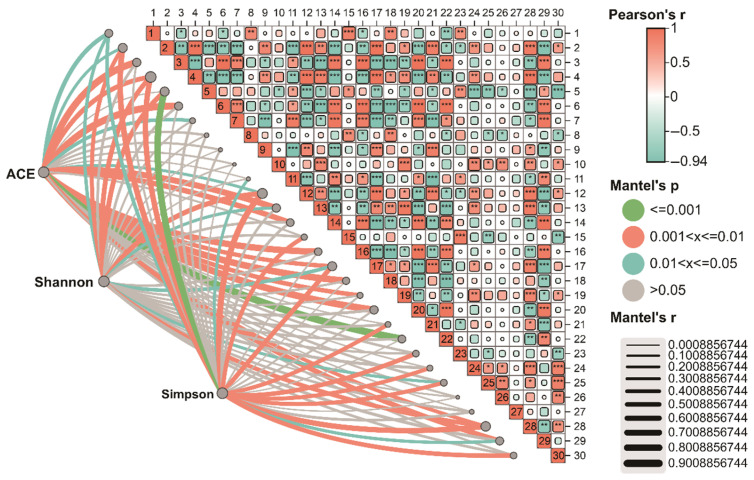
Mantel correlation analysis based on bacterial communities. 1: *Pseudomonadota*, 2: Actinomycetota, 3: *Acidobacteriota*, 4: Chloroflexota, 5: *Myxococcota*, 6: *Candidatus*_*Rokubacteria*, 7: *Gemmatimonadota*, 8: Bacteroidota, 9: *Bacillota*, 10: Planctomycetota, 11: *Luteitalea*, 12: *Anaerolinea*, 13: *Steroidobacter*, 14: *Gemmatimonas*, 15: *Sphingomonas*, 16: *Nitrospira*, 17: *Thermopolyspora*, 18: *Hyphomicrobium*, 19: *Streptomyces*, 20: *Candidatus*_*Solibacter*, 21: *Aggregatilinea*, 22: *Candidatus*_*Koribacter*, 23: pH, 24: OM, 25: TN, 26: TP, 27: TK, 28: AN, 29: AP, 30: AK. * (0.01 ≤ *p* < 0.05), ** (0.001 ≤ *p* < 0.01), *** (*p* < 0.001).

In the bacterial community, AP was significantly positively correlated with *Acidobacteriota*, *Myxococcota*, *Candidatus Rokubacteria*, *Gemmatimonadota*, *Luteitalea*, *Gemmatimonas*, *Nitrospira*, *Candidatus Solibacter*, and *Candidatus Koribacter*. Conversely, it showed significant negative correlations with Actinomycetota, Chloroflexota, *Bacillota*, *Anaerolinea*, *Steroidobacter*, *Thermopolyspora*, *Hyphomicrobium*, *Streptomyces*, and *Aggregatilinea*. Soil pH was significantly positively correlated with *Pseudomonadota*, *Myxococcota*, *Luteitalea*, and *Sphingomonas*, but significantly negatively correlated with Actinomycetota, *Anaerolinea*, and *Aggregatilinea* (Figure 7).

### 3.5. Correlation Analysis of α Diversity with Key Microorganisms and Soil Chemical Properties

To further elucidate the ecological relationships, this study performed Mantel analysis to examine the associations between the α-diversity indices (ACE, Shannon, Simpson) of eukaryotic and bacterial communities with key environmental factors and microbial taxa.

For the eukaryotic community, the ACE index exhibited significant correlations with several bacterial phyla, including *Pseudomonadota*, Actinomycetota, *Acidobacteriota*, Chloroflexota, *Myxococcota*, *Candidatus Rokubacteria*, and Bacteroidota, as well as with the bacterial genera *Sphingomonas*, *Nitrospira*, *Hyphomicrobium*, and *Candidatus Koribacter*. It was also significantly linked to the environmental factors matter (OM), TK, and AP. The Shannon and Simpson indices of eukaryotes showed significant correlations with the bacterial phylum Chloroflexota, the genus *Gemmatimonas*, as well as *Steroidobacter* and *Streptomyces*. These diversity indices were significantly influenced by soil pH, OM, TN, TK, and AP. Additionally, the Simpson index showed a significant correlation with AN (Figure 6).

Within the bacterial community, the ACE index was significantly correlated with multiple bacterial phyla and genera, as well as with AN and AP. The Shannon index demonstrated significant associations with the phyla *Pseudomonadota*, Actinomycetota, and Chloroflexota, the genera *Anaerolinea*, *Steroidobacter*, and *Thermopolyspora*, and the environmental factors TN and AN. The Simpson index was significantly correlated with a broader range of taxa, including the phyla *Pseudomonadota*, Actinomycetota, Chloroflexota, and *Myxococcota*, the genera *Luteitalea*, *Anaerolinea*, *Sphingomonas*, *Thermopolyspora*, and *Aggregatilinea*, and multiple environmental factors (pH, OM, TN, AN, AP, and AK) (Figure 7).

## 4. Discussion

### 4.1. Effects of Different Treatments on Soil Chemical Properties

Cultivation practices and soil amendments are key agronomic strategies for regulating soil chemical properties and the microbial environment. This study investigated the effects of rotary tillage, CaCN_2_, and organic fertilizer application on soils subjected to *Morchella* continuous cropping. The results indicate that different treatments significantly altered soil properties through distinct mechanisms. Unlike previous reports suggesting that long-term rotary tillage may cause compaction in deeper soil layers [10,11], the tillage practice in this study likely improved near-surface nutrient distribution and transformation primarily through physical disturbance. In this study, it was observed that although rotary tillage alone may increase soil pH to some extent [16], the combined application of CaCN_2_ resulted in a significant decrease in soil pH. Calcium cyanamide is generally considered to have alkaline or neutralizing effects; however, the acidification observed in this study may be attributed to the following factors. First, cyanamide (H_2_CN_2_) produced during the hydrolysis of calcium cyanamide is further converted into urea, which, upon microbial nitrification, releases H^+^ and leads to a decline in soil pH. Second, the application of calcium cyanamide promotes the accumulation of ammonium nitrogen, which also generates acidity during the nitrification process [17]. The application of CaCN_2_ significantly increased the contents of OM, TN, AN, AK, and tTP. These changes may result not only from the direct nutrient input provided by CaCN_2_ but also from its regulatory effects on microbial activity. For instance, the inhibitory effect of CaCN_2_ decomposition products on certain microbial groups may slow the decomposition of organic matter, thereby promoting its accumulation. In addition, CaCN_2_ application may indirectly enhance nutrient availability by altering soil physicochemical properties [18]. Notably, when CaCN_2_ was applied in combination with organic fertilizer, the contents of total nitrogen and available nitrogen in the soil were lower than those under CaCN_2_ application alone. This phenomenon may be associated with enhanced microbial activity under high nitrogen inputs, which could accelerate the mineralization of organic nitrogen [19,20].

In summary, rotary tillage, CaCN_2_, and organic fertilizer application all improved soil Chemical properties to varying degrees. The observed differences in nutrient profiles among treatments are likely attributable to their differential impacts on the structure and function of the soil microbial community. Notably, the soil organic matter content under the MPX treatment was slightly lower than that under the MPD treatment (Figure 1B). This phenomenon may be associated with a “priming effect” induced by the addition of organic fertilizer, whereby the input of fresh organic substrates stimulates microbial activity and accelerates the decomposition and mineralization of native soil organic matter [21]. In addition, the available phosphorus content in the CK was significantly higher than that in the other three treatments (Figure 1H). The initial available phosphorus content of the experimental soil was approximately 100 mg·kg^−1^, which is relatively high and may be associated with phosphorus accumulation during long-term continuous cropping. Rotary tillage (XGX) may have altered the distribution and transformation of phosphorus within the plow layer, thereby reducing its availability. The application of calcium cyanamide may have further decreased phosphorus availability by modifying soil pH or promoting the formation of calcium phosphate precipitates through interactions with calcium ions. It should be noted that the experimental design of this study did not include treatments with calcium cyanamide alone, organic fertilizer alone, or their binary combination. Therefore, the interaction effects between rotary tillage and soil amendments could not be directly quantified, and the independent contributions of each factor to soil properties could not be clearly distinguished. Future studies should incorporate a more comprehensive set of treatment combinations, where feasible, to further elucidate the synergistic and antagonistic mechanisms among different soil management practices [22].

### 4.2. Effects of Different Treatments on Soil Microbial Communities

Soil microorganisms, as pivotal agents of terrestrial ecosystem function, play a central role in driving organic matter decomposition and maintaining soil health. Their community structure is widely recognized as a key indicator of soil quality [23,24]. The findings of this study demonstrate that different soil management practices significantly altered the composition and diversity of the soil microbiota. Rotary tillage alone increased the ACE index of both eukaryotic and bacterial communities, indicating a potential positive effect on microbial richness. Due to its functions in soil disinfection and microbial community regulation [25], the application of CaCN_2_ in combination with rotary tillage significantly decreased the ACE and Shannon indices of the eukaryotic community while increasing the Simpson index, suggesting a potential simplification of community structure. This change may be associated with the inhibitory effects of CaCN_2_ on certain microbial groups; however, the specific taxa affected require further clarification based on subsequent analyses of community composition. In contrast, the MPD treatment resulted in a decrease in the Simpson index of the bacterial community, indicating a differential effect on bacterial evenness.

The addition of organic fertilizer provides a rich source of nutrients and energy for microorganisms, facilitating the activation of beneficial groups and enhancing community complexity and stability [26,27,28]. In this study, the integrated application of organic fertilizer (MPX treatment) mitigated the inhibitory effect of CaCN_2_ on eukaryotic diversity, as evidenced by an increased Shannon index and a decreased Simpson index compared to the MPD treatment. This indicates that organic fertilizer can alleviate the suppression of eukaryotic diversity and improve community evenness. Soil eukaryotes, encompassing fungi and protists, contribute to system stability through ecological interactions such as predation and competition, and their increased diversity can help suppress pathogen proliferation [29,30]. Regarding the bacterial community, the MPD and MPX treatments significantly increased the Shannon index and decreased the Simpson index, indicating a promotion of bacterial diversity. Notably, eukaryotic and bacterial communities exhibited markedly different responses to CaCN_2_ application. CaCN_2_ significantly reduced the α-diversity of the eukaryotic community, while exerting a certain promoting effect on bacterial diversity. This discrepancy may be attributed to differences in sensitivity to CaCN_2_ decomposition products (e.g., cyanamide). Eukaryotic microorganisms, particularly fungi, may be more sensitive to cyanamide, whereas some bacterial taxa may possess greater tolerance or the ability to utilize CaCN_2_-derived compounds as nutrient sources [31]. In addition, differences in ecological niches and functional roles between these two groups may also contribute to their distinct responses to environmental disturbances. Enhanced bacterial diversity is often associated with reduced pathogen incidence [32,33], as it increases functional redundancy, community resilience, and participation in key processes like nutrient cycling and litter decomposition [34,35,36].

At the phylum level, the treatments differentially affected microbial distributions. Rotary tillage increased the relative abundance of *Basidiomycota*, fungi typically involved in organic matter decomposition and plant symbiosis [37]. The addition of CaCN_2_ significantly reduced its abundance, likely due to its general fungistatic effect. Notably, all three treatments effectively reduced the relative abundance of *Mucoromycota*, a phylum containing several pathogenic species associated with crop rot [38], thereby potentially lowering the risk of soil-borne disease.

Notably, all three treatments in this study significantly reduced the relative abundance of *Olpidium*. Fungi of the genus *Olpidium* are obligate vectors of various soil-borne viruses; their zoospores can infect plant roots and facilitate virus transmission, and they are considered important pathogen-associated taxa in continuous-cropping systems of many crops36. In the *Morchella* continuous-cropping soil examined in this study, the decline in the abundance of this genus may contribute to a reduction in the risk of soil-borne diseases. Among the treatments, MPX showed the most pronounced effect. This suggests that the optimization of soil structure by rotary tillage, the disinfecting function of calcium cyanamide, and the stimulatory effect of organic fertilizer on beneficial microorganisms jointly contribute to the effective suppression of *Olpidium* [39].

For *Morchella* itself, rotary tillage significantly increased its relative abundance, while the addition of CaCN_2_ had an inhibitory effect. However, this suppression was partially alleviated when organic fertilizer was co-applied. These results suggest that rotary tillage may influence the soil microenvironment by altering soil physical structure, thereby creating favorable conditions for *Morchella* growth; however, the underlying mechanisms require further investigation. The strong oxidative properties and chemical regulatory effects of calcium cyanamide may transiently inhibit mycelial development, whereas the addition of organic fertilizer may partially alleviate this inhibition by supplying labile organic matter and buffering chemical stress. The primary objective of this study was to investigate the effects of rotary tillage and soil amendments on soil chemical properties and microbial communities in continuously cultivated *Morchella* soils. Accordingly, the analysis focused on soil physicochemical characteristics and microbial community features, and agronomic traits such as *Morchella* yield were not directly measured. This represents a limitation of the study, which will be addressed in future research. At the bacterial phylum level, *Pseudomonadota*, Actinomycetota, and Bacteroidota increased in relative abundance across treatments. *Pseudomonadota*, containing major phosphate-solubilizing families, likely contributed to phosphorus mineralization [40]. Actinomycetota are widely distributed in soils, and some taxa possess antimicrobial activity and the capacity to decompose organic matter, thereby playing important roles in maintaining soil health [41,42,43]. In this study, this phylum exhibited the highest abundance under the MPX treatment, which may be associated with the increased availability of organic substrates and the improved microenvironment provided by this treatment. The increase in Bacteroidota, involved in carbon cycling [44], under tillage and organic fertilizer treatments may be linked to enhanced availability of organic substrates.

At the genus level, the relative abundances of functional bacteria such as *Anaerolinea*, *Steroidobacter*, and *Hyphomicrobium* were differentially affected by the treatments. For instance, *Anaerolinea* was promoted by all treatments, whereas *Steroidobacter* increased in the XGX and MPD treatments but was inhibited in MPX. *Hyphomicrobium* increased in MPX but decreased in MPD. These results underscore that different soil management practices achieve selective regulation of specific functional groups by modifying nutrient availability and the soil microenvironment, ultimately influencing soil ecosystem health and the sustainability of *Morchella* production.

It should be noted that soil samples in this study were collected one month after treatment, and the observed changes in microbial communities therefore mainly reflect short-term responses. In addition, treatments with calcium cyanamide alone, organic fertilizer alone, and their factorial combinations were not included, which limits the ability to disentangle the individual contributions of each factor. Consequently, the effects observed under the MPX treatment represent the combined influence of rotary tillage, calcium cyanamide, and organic fertilizer.

### 4.3. Effects of Soil Chemical Properties and Key Microbial Groups on Microbial Community Diversity

Environmental factor analysis indicated that available nitrogen (AN) and available potassium (AK) were the key drivers of variation in the eukaryotic community, whereas available phosphorus (AP) and pH primarily influenced the structure of the bacterial community (Figure 5). Mantel analysis revealed significant associations between soil Chemical properties and the community diversity of both eukaryotes and bacteria. The ACE index of the eukaryotic community and the Simpson index of the bacterial community were notably correlated with soil pH. In this study, soil pH differed significantly among treatments (Figure 1A). The XGX treatment had a significant effect on pH, and the application of calcium cyanamide also resulted in a decrease in pH (Figure 1A). Soil microorganisms are highly sensitive to changes in pH [45]; even fluctuations of less than one unit can substantially reshape the microbial habitat [46]. Further analysis indicated that the ACE, Shannon, and Simpson indices of the eukaryotic community were linked to the contents of AN and AK. In contrast, the alpha diversity indices of the bacterial community were primarily associated with AN. This suggests that AN and AK are closely associated with the diversity and composition of eukaryotic microbial communities, potentially through indirect effects on the abundance of specific key microbial taxa. AN and AK are key nutrients for the growth and metabolism of eukaryotic microorganisms. AN, as a readily available nitrogen source, directly supports microbial protein synthesis and cell proliferation [47,48]. AK plays an important role in osmotic regulation and enzyme activation [49]. Eukaryotic microorganisms, particularly fungi, generally exhibit higher nutrient demands, and their hyphal growth is highly sensitive to the spatial distribution of soil nutrients. Therefore, variations in AN and AK may regulate eukaryotic community structure by influencing nutrient availability and acquisition efficiency [50,51]. In contrast, many bacterial taxa possess greater metabolic versatility and nutrient acquisition capabilities, such as biological nitrogen fixation and mineral solubilization, and can utilize a wider range of carbon and nitrogen sources [52,53]. Consequently, bacterial communities tend to be less sensitive to fluctuations in AN and AK compared with eukaryotic microorganisms.

The Shannon index, as a composite indicator reflecting species richness and evenness, showed significant correlations with multiple microbial taxa in this study. Among eukaryotes, taxa such as *Chytridiomycota* and Discosea are known to participate in organic matter decomposition and the regulation of microbial food webs [54,55]. Among bacteria, groups such as Pseudomonadota and Actinomycetota play important roles in nutrient cycling and pathogen suppression [56,57,58]. The significant associations between these taxa and the Shannon index suggest that they may contribute to the maintenance of soil microbial community stability. It should be noted that the correlation analysis in this study involved both phylum- and genus-level taxa. Differences in taxonomic resolution may influence the interpretation of the results; therefore, future studies should focus on a consistent taxonomic level to improve methodological robustness.

## 5. Conclusions

This study systematically evaluated the effects of different agronomic practices on the improvement of soils under continuous morel cultivation. The main conclusions are as follows: the MPD treatment (calcium cyanamide combined with rotary tillage) exhibited relatively strong effectiveness in improving soil chemical properties, simultaneously reducing soil pH while increasing OM, TN, AN, AP, and TP. In contrast, the XGX treatment, although capable of altering soil physical structure, demonstrated limited efficacy in elevating soil nutrient levels. All treatments significantly reshaped the structure of the soil microbial community. Although the XGX treatment enhanced eukaryotic diversity, it failed to effectively suppress the growth of pathogenic fungi. The MPD treatment substantially reduced the abundance of pathogenic fungi such as *Olpidium* through the disinfectant action of calcium cyanamide, although it concurrently exerted an inhibitory effect on overall eukaryotic diversity. The integrated MPX treatment optimally suppressed pathogens while maintaining higher microbial diversity, thereby achieving a more balanced and beneficial regulation of the soil microbiome. Analysis of environmental drivers identified AN and AK as the primary factors shaping the eukaryotic community, whereas AP and pH were the key determinants of bacterial community structure. These factors mediated their effects by modulating the relative abundance of beneficial bacterial phyla and pathogenic fungal groups. Mantel analysis further indicated that soil nutrient status was closely associated with microbial taxa such as *Anaerolinea*, *Steroidobacter*, and *Hyphomicrobium*. In summary, the MPD treatment showed relatively strong performance in improving soil chemical properties and suppressing pathogens, whereas the MPX treatment was relatively more effective in optimizing microbial community structure, promoting the recovery of microbial diversity, and facilitating the establishment of beneficial microbial populations. These two treatments demonstrated distinct advantages in soil chemical improvement and microbial community regulation, respectively, providing alternative technical approaches for the ecological management of continuous-cropping obstacles in *Morchella* cultivation.

## Data Availability

The original contributions proposed in the study are included in the article, and further inquiries can be directed to the corresponding authors.

## Supplementary Materials

The following supporting information can be downloaded at: https://www.mdpi.com/article/10.3390/biology15090674/s1. Table S1: Statistics of soil genome DNA sequencing data; Table S2: Statistical Summary of Forward Selection Results from RDA of Eukaryotic Communities and Environmental Factors; Table S3: Contribution and Significance Analysis of Environmental Factors to Variation in Eukaryotic Communities; Table S4: Statistical Summary of RDA Forward Selection Results for Bacterial Communities and Environmental Factors; Table S5: Statistical Summary of RDA Forward Selection Results for Bacterial Communities and Environmental Factors; Figure S1: Snapshots of the morel cultivation process. Rotary tillage scene (A); Sampling scene (B); Scene of placing nutrient bags (C); Fruiting scene (D). Figure S2: Venn diagram of eukaryotic microbial communities (A) and bacterial communities (B); Figure S3: Multi-group comparative analysis Eukaryotic phylum level (A), eukaryotic genus level (B), bacterial phylum level (C), bacterial genus level (D).
