# Supplementary material for: Effects of Rotary Tillage and Fertilization on Chemical Properties and Microbial Communities of Soil Under Continuous Morchella Mushroom Cultivation
Source: Biology (Basel). 2026 Apr 24;15(9):674. doi: 10.3390/biology15090674 (PMC13162986; doi:10.3390/biology15090674)
Supplement: Supplementary file 1 [file biology-15-00674-s001.zip › biology-4229293-supplementary.pdf]

Effects of Rotary Tillage and Fertilization on Chemical Properties and Microbial Communities of Soil Under Continuous *Morchella* Mushroom Cultivation

Wei Qi [1,†], Litao Lü [1,†], Kai Huang [2,3], Jianzhao Qi [3,4], MingLei Li [2,3], Mingwen Shi [1] and Hong Wang [1,*]

1. Institute of Edible Fungi Liaoning Academy of Agricultural Sciences, Shen yang 110000, China
2. School of Soil and Water Conservation Science and Engineering, Northwest A&F University, Yangling 712100, China
3. Center of Edible Fungi, Northwest A&F University, Yangling 712100, China
4. Shaanxi Key Laboratory of Natural Products & Chemical Biology, College of Chemistry & Pharmacy, Northwest A&F University, Yangling 712100, China

† These authors contributed equally to this work.
* Correspondence: wang45852676@126.com；Tel： +8613840347121

Table S1: Statistics of soil genome DNA sequencing date.

Samples	Raw reads	Raw base (bp)	Clean reads	Clean base(bp)	Percent in raw reads (%)	Percent in raw bases (%)	Raw Q30 (%)	Clean Q30 (%)
XGX_1	7.68E+07	1.16E+10	7.51E+07	1.13E+10	97.81	97.45	92.98	94.15
XGX_2	6.59E+07	9.96E+09	6.47E+07	9.73E+09	98.11	97.74	93.12	94.24
XGX_3	7.39E+07	1.12E+10	7.24E+07	1.09E+10	97.89	97.54	93.03	94.15
MPD_1	7.32E+07	1.10E+10	7.16E+07	1.08E+10	97.80	97.42	92.77	93.94
MPD_2	7.06E+07	1.07E+10	6.89E+07	1.04E+10	97.54	97.15	92.5	93.77
MPD_3	7.65E+07	1.15E+10	7.47E+07	1.12E+10	97.68	97.28	92.71	93.95
MPX_1	6.91E+07	1.04E+10	6.76E+07	1.02E+10	97.78	97.41	92.82	93.99
MPX_2	8.08E+07	1.22E+10	7.90E+07	1.19E+10	97.70	97.32	92.78	94
MPX_3	7.55E+07	1.14E+10	7.37E+07	1.11E+10	97.58	97.21	92.75	93.99
CK1	7.38E+07	1.11E+10	7.25E+07	1.09E+10	98.22	97.90	93.18	94.3
CK2	7.67E+07	1.16E+10	7.52E+07	1.13E+10	98.09	97.75	92.88	94.08
CK3	7.16E+07	1.08E+10	7.02E+07	1.06E+10	97.97	97.63	93.07	94.2

Table S2: Statistical Summary of Forward Selection Results from RDA Analysis of Eukaryotic Communities and Environmental Factors.

Analysis 'Interactive-forward-selection', step 'Forward Selection'
Method: RDA
Total variation is 0.00000, explanatory variables account for 96.2%,
(adjusted explained variation is 86.0%)
Summary Table:

Statistic	Axis 1	Axis 2	Axis 3	Axis 4
Eigenvalues	0.8756	0.0561	0.0194	0.0098
Explained variation (cumulative)	87.56	93.17	95.11	96.09
Pseudo-canonical correlation	0.9834	0.9889	0.9071	0.9652
Explained fitted variation (cumulative)	91.04	96.87	98.89	99.91

Table S3: Contribution and Significance Analysis of Environmental Factors to Variation in Eukaryotic Communities.

Analysis 'Interactive-forward-selection', step 'Forward Selection' Forward Selection Results:				
Name	Explains %	Contribution %	pseudo-F	P
AK（mg/kg）	54.8	57	12.1	0.006
AN（mg/kg）	33.8	35.2	26.8	0.006
OM	3.6	3.7	3.6	0.016
TK	1.9	2	2.3	0.096
pH	1.5	1.6	2.1	0.15
TP	0.3	0.3	0.4	0.724
TN	0.2	0.2	0.2	0.888
AP（mg/kg）	<0.1	<0.1	<0.1	0.968

Table S4: Statistical Summary of RDA Forward Selection Results for Bacterial Communities and Environmental Factors.

Analysis 'Interactive-forward-selection', step 'Forward Selection'
Method: RDA
Total variation is 0.01898, explanatory variables account for 99.0%
(adjusted explained variation is 96.4%)
Summary Table:

Statistic	Axis 1	Axis 2	Axis 3	Axis 4
Eigenvalues	0.7424	0.2305	0.0151	0.001
Explained variation (cumulative)	74.24	97.3	98.81	98.91
Pseudo-canonical correlation	0.9981	0.9932	0.93	0.8543
Explained fitted variation (cumulative)	74.99	98.27	99.79	99.9

Table S5: Statistical Summary of RDA Forward Selection Results for Bacterial Communities and Environmental Factors.

Analysis 'Interactive-forward-selection', step 'Forward Selection' Forward Selection Results:				
Name	Explains %	Contribution %	pseudo-F	P
AP	62.2	62.8	16.4	0.002
pH	25.2	25.5	18	0.002
AN	8.6	8.7	17.3	0.002
AK	1.6	1.7	5	0.024
TK	0.8	0.8	3.3	0.076
OM	0.2	0.2	0.9	0.466
TP	0.2	0.2	0.8	0.526
TN	<0.1	<0.1	0.2	0.872

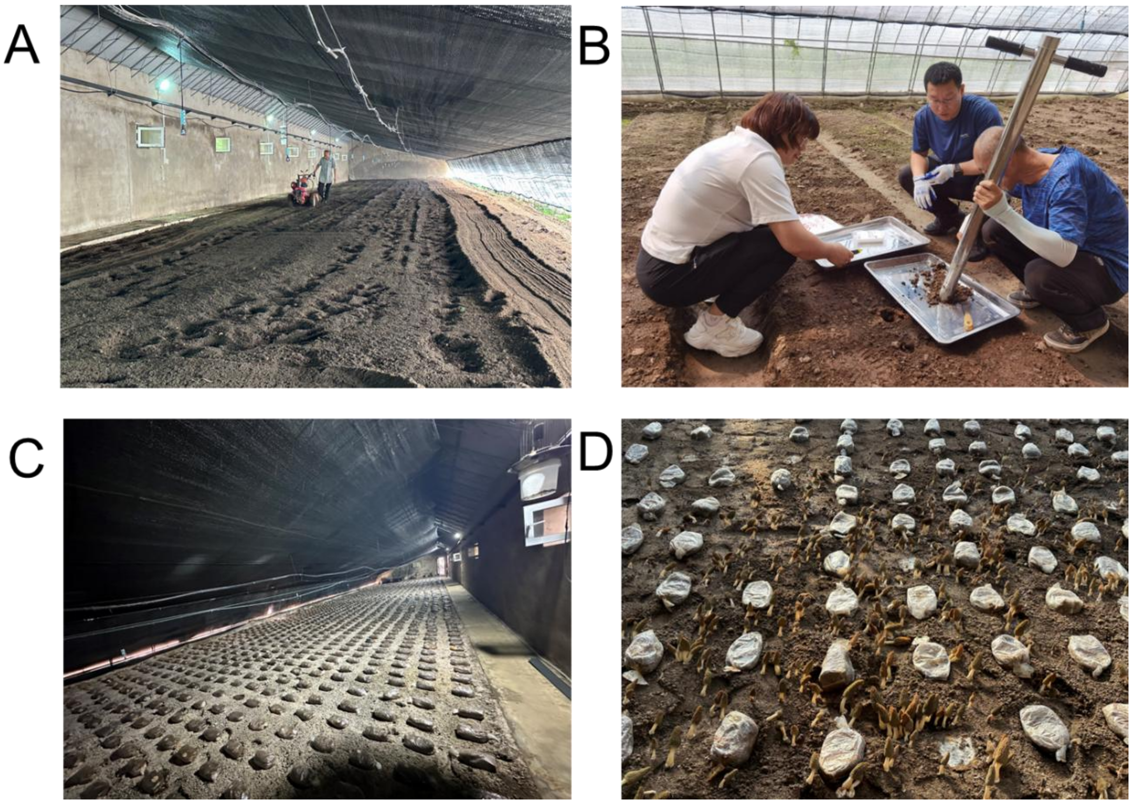

Figure S1. Snapshots of the morel cultivation process. Rotary tillage scene (A); Sampling scene (B); Scene of placing nutrient bags (C); Fruiting scene (D).

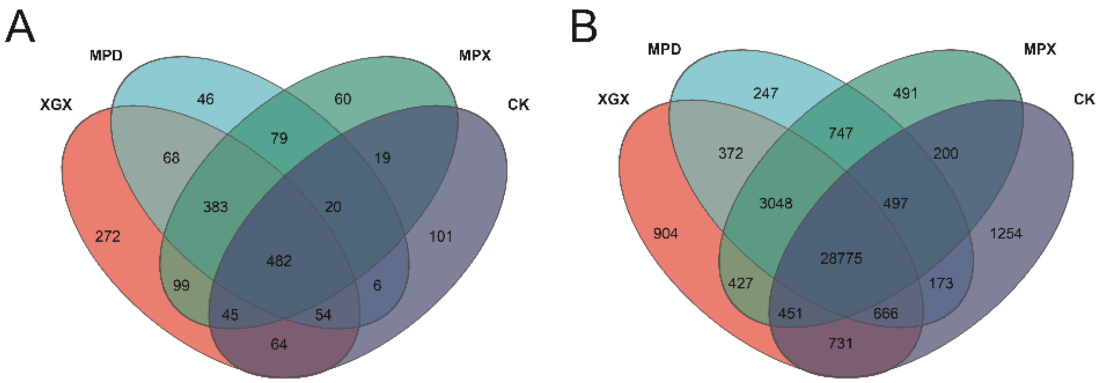

Figure S2. Venn diagram of eukaryotic microbial communities (A) and bacterial communities (B)

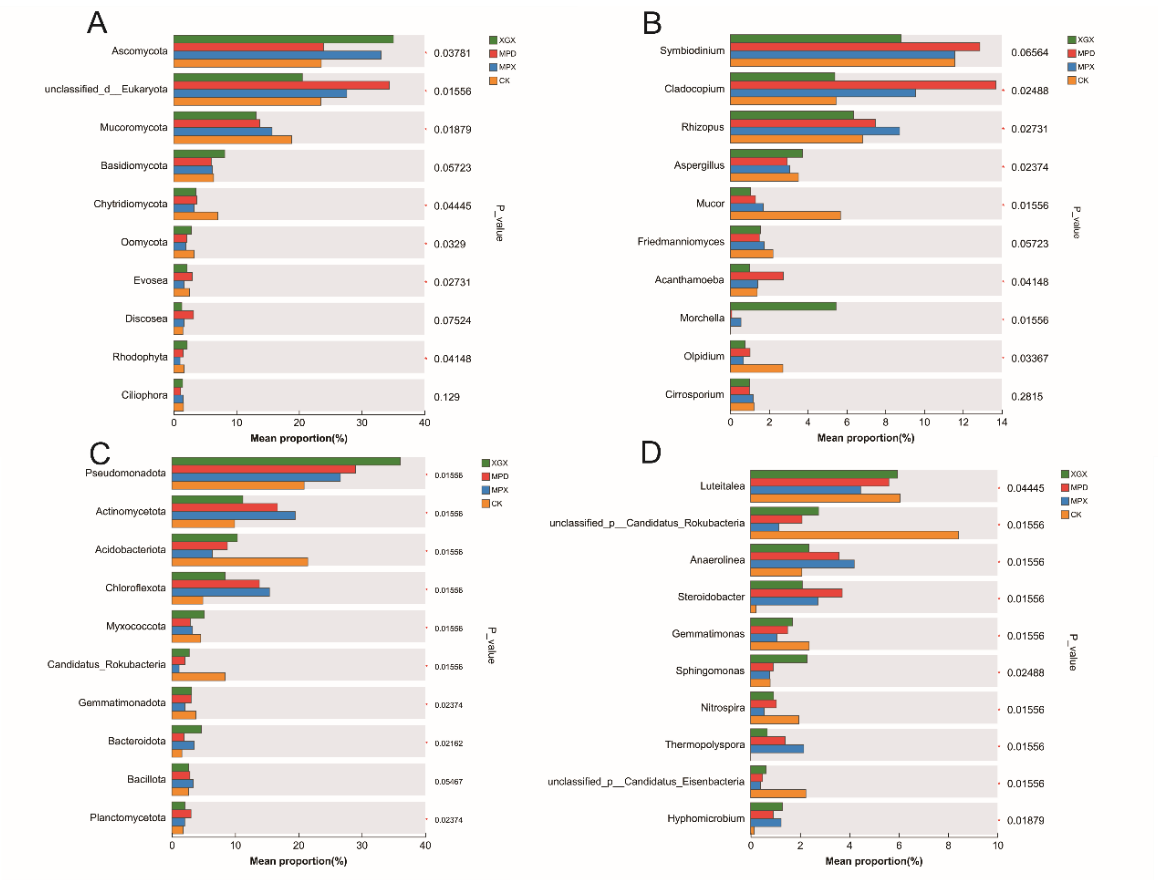

Figure S3. Multi-group comparative analysis Eukaryotic phylum level (A), eukaryotic genus level (B), bacterial phylum level (C), bacterial genus level(C).
